# Neural correlates of two different types of extinction learning in the amygdala central nucleus

**DOI:** 10.1038/ncomms12330

**Published:** 2016-08-17

**Authors:** Mihaela D. Iordanova, Mickael L. D. Deroche, Guillem R. Esber, Geoffrey Schoenbaum

**Affiliations:** 1 National Institute on Drug Abuse Intramural Research Program, Cellular Neurobiology Research Branch, Behavioral Neurophysiology Research Section, 251 Bayview Boulevard, Baltimore, Maryland 21224, USA; 2 Department of Anatomy and Neurobiology, University of Maryland, School of Medicine, 20 Penn Street, Baltimore, Maryland 21201, USA; 3 Department of Psychology and Centre for Studies in Behavioral Neurobiology, Concordia University, 7141 Sherbrooke West, Montreal, Quebec, Canada H4B 1R6; 4 Center for Research on Brain, Language, and Music, McGill University, 3640 Rue de la Montagne, Montreal, Quebec, Canada H3G 2A8; 5 Solomon H. Snyder Department of Neuroscience, the Johns Hopkins Univeristy, Baltimore, Maryland 21287, USA

## Abstract

Extinction is a fundamental form of memory updating in which one learns to stop expecting an event that no longer occurs. This learning ensues when one experiences a change in environmental contingencies, that is, when an expected outcome fails to occur (simple extinction), or when a novel inflated expectation of a double outcome (overexpectation) is in conflict with the real outcome, and is a process that has been linked to amygdala function. Here, we show that in rats, the same neuronal population in the amygdala central nucleus updates reward expectancies and behaviour in both types of extinction, and neural changes in one paradigm are reflected in the other. This work may have implications for the management of addiction and anxiety disorders that require treatments based on the outcome omission, and disorders such as obesity that could use overexpectation, but not omission strategies.

## Abstract (web summary)

There are two forms of extinction learning, which are vital for adaptive behaviour: simple extinction, when an expected outcome fails to occur, and overexpectation, when an exaggerated expectation is in conflict with the actual outcome. Iordanova *et al.* show that both forms of extinction learning have a common neural substrate in the amygdala.

## Graphical abstract

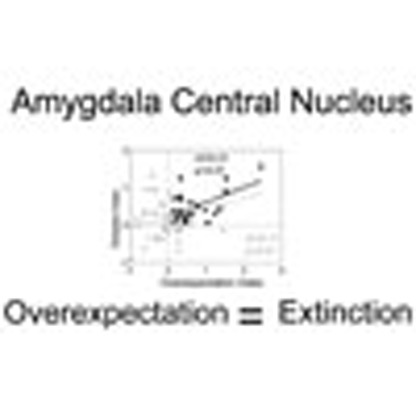

The accurate representation of predictive relationships between events in the environment is critical for adaptive behaviour. In an ever-changing world, the brain must constantly update its predictions, so that costly resources are no longer spent in attempts to acquire previously offered but now unavailable rewards. Such a reduction in the predictive potency of a cue can be achieved by holding the expectation constant and manipulating the delivery of the outcome—that is, simple extinction^1^—or by holding outcome delivery constant and manipulating the expectation—that is, overexpectation^2^,^3^. In simple extinction, the delivery of an expected outcome is omitted, hereby termed ‘extinction by omission'. In overexpectation, the previously expected outcome continues to be delivered, but suddenly fails to meet an inflated expectation^4^. In omission, past realities are explicitly challenged, whereas in overexpectation one's novel expectations about realities go unmet. What is particularly interesting about the latter type of extinction learning is that it serves to undermine a well-established association with an outcome even in the presence of the outcome itself. As such, overexpectation offers a behavioural way to reduce the predictive power of a cue in circumstances when omitting the delivery of a reinforcer is not an option, for example, in treating obesity.

Omission and overexpectation represent two distinct conditions that promote extinction learning and are underscored by the same theoretical framework^2^,^3^,^5^. Yet, it remains unknown whether the real-time processing of each paradigm is done by common or independent neuronal populations. It would be striking to find that an overlapping population of cells processes omission and overexpectation despite their procedural differences. A shared population suggests that the two procedures could be thought of as interchangeable in some therapeutic settings, in which case they may work together to provide a stronger extinction effect, or may lead to learning interference. Furthermore, understanding the representation of these different types of extinction in the brain poses novel questions about the neural and behavioural processes that govern the renewal or recovery from extinction learning^2^,^3^,^6^. This may help the development of clinical treatments that effectively reduce the predictive power or acquired valence of cues such as exposure therapy or abstinence.

To shed light on this issue, we recorded single-unit activity from the amygdala central nucleus (CN) in rats during a single task in which expectations were violated either by omitting expected rewards (simple extinction)^1^ or by generating overexpectation of rewards^4^. The CN is a key candidate for processing learning under these conditions. Neural activity in the CN signals when rewards are omitted^7^, and damage to the CN disrupts extinction learning when rewards are omitted^8^ or overexpected^9^,^10^. Similar findings have been reported in aversive settings^11^,^12^ (but also see ref. 13). These findings point to the possibility that CN neurons track outcome expectations whenever those expectations are reduced or use changes in outcome expectations to reinstate cue salience that has suffered losses^10^. In either case, if the process is occurring through a mechanism that is common to both kinds of extinction, then we would predict the change in neural firing to be correlated between the two conditions, with the activity of a common neural population predicting the decline in behaviour that is characteristic of both phenomena. If, on the other hand, the two types of extinction operate on distinct representations, at least at the level of CN, then we would expect to find separate sets of CN neurons tracking outcome expectations and predicting behaviour for each condition. Our findings show that omission and overexpectation are processed by an overlapping set of CN cells, and this population predicts the decline in behavioural responding that is characteristic of extinction learning.

## Results

### Design

**Figure 1:**
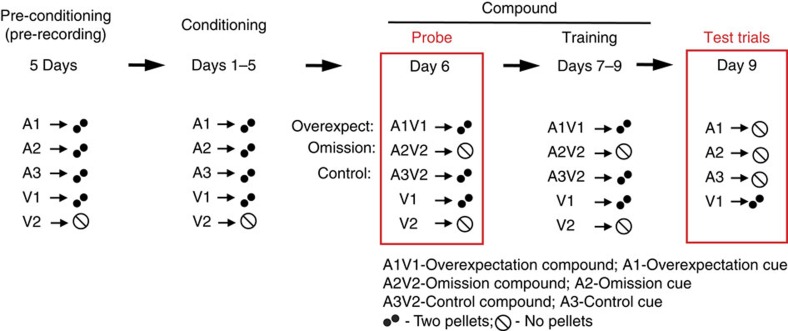
Behavioural design. A1–3 denote auditory cues, V1–2 denote visual cues. A1V1—overexpectation compound. A2V2—omission compound. A3V2—control compound.

The behavioural design used in this study is illustrated in Fig. 1. Rats were trained across the following three stages: pre-conditioning, conditioning and compound, and tested immediately following the end of the last compound day. During the pre-conditioning (before any surgical procedures and thus before neural recordings began) and conditioning (following electrode implantation) sessions rats were trained to expect two grape-flavoured sucrose pellets following the presentation of each of three auditory cues (A1–3: clicker, white noise, or tone,counterbalanced) and following one, but not another, visual cue (V1 and V2: flashing or steady light, counterbalanced). Extinction learning was elicited and assessed during the subsequent compound phase. During this phase, two critical extinction conditions were created which allowed us to manipulate either the expectation (that is, extinction by overexpectation) or the actual delivery (that is, extinction by omission) of reward and to assess their effect on behaviour. The overexpectation condition was created by presenting two previously reinforced stimuli (A1V1: one auditory, one visual) in compound. This led to a prediction of additional reward; yet this compound was reinforced with the same two pellets delivered on trials during conditioning. The omission condition was created by presenting one of the remaining auditory cues in compound with the visual cue (A2V2) that was not previously reinforced, resulting in the prediction of two pellets, but no pellets were delivered. Although they are procedurally different, both overexpectation and omission generate conditions in which fewer-than-expected rewards are delivered. This discrepancy generates a negative prediction error and should result in a similar reduction in outcome expectancy to the target cues. These conditions were compared with a control condition in which the predicted rewards were identical to the delivered rewards, and therefore there was no prediction error, thus yielding no change in the association between the control cue and reward. The control condition was created by presenting the third auditory cue in compound with the previously non-reinforced visual cue (A3V2), much like the omission compound, yielding an expectation of two pellets. Importantly, unlike the omission compound, the control compound was reinforced with the expected two pellets. In addition to the compound presentations, the two visual cues continued to be presented individually and were either reinforced or not reinforced in the same manner as they were during conditioning. This was done to direct and maximize any changes in reward expectation that resulted from overexpectation training towards the target auditory cue.

As the overexpectation condition necessarily required the compounding of two previously reinforced cues, it also created the possibility that behavioural and neural responding would be influenced by the compound novelty or the integration of audio–visual information. Omission and control conditions that also use novel audio–visual compounds control for the influence of these extraneous variables. This optimizes the direct comparison of responding (behavioural or neural) between the three compounds during the critical compound probe phase.

To confirm that the overexpectation and omission conditions resulted in reduction in the association between the target cues and reward, behavioural and neural responding was examined during non-reinforced presentation of the overexpectation and omission cues on test. The data presented below were obtained from 9 rats (total of 16 sessions) that exhibited behavioural evidence of overexpectation and extinction during this test. The 6 rats (total of 8 sessions) that failed to show these behavioural effects, also did not exhibit the neural profile described below (see Supplementary Note 1 and Supplementary Fig. 1).

### Pre-conditioning and conditioning phases

**Figure 2:**
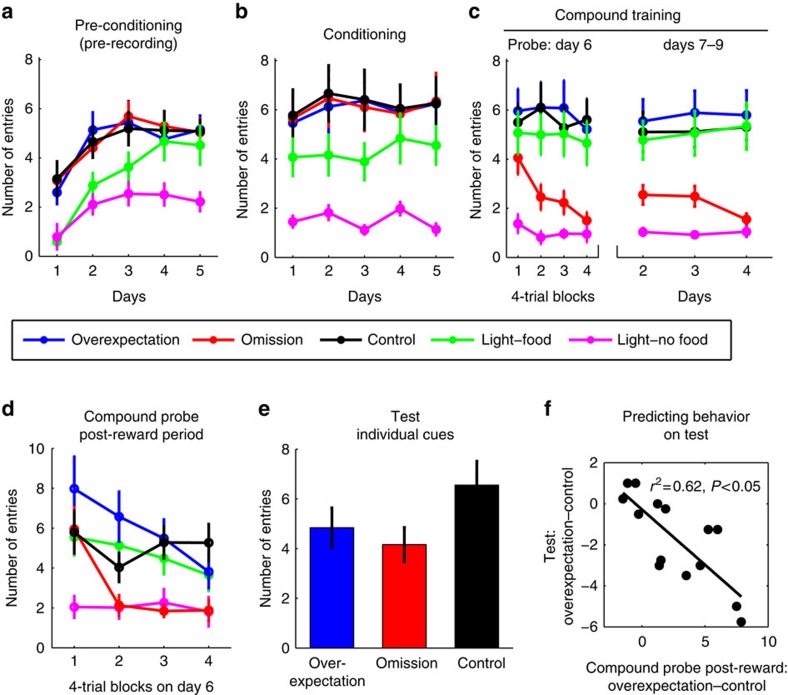
Behavioural data. (**a**) Pre-Conditioning. A two-way repeated measures analysis of variance (ANOVA) found a main effect of cue (F(4,32)=12.2, *P*<0.05), an effect of session (F(4,32)=6.95, *P*<0.05) and an interaction (F(16,128)=1.8, *P*<0.05). *Post hoc* testing revealed no differences in responding among the auditory cues across days (all *q*<1, *P*>0.55), no difference between the visual cues on days 1–3 (_max_ *q*=2.3, *P*>0.05) but a difference on the last 2 days (days 4 and 5, min *q*=7.9, *P*<0.05). (**b**) Conditioning. A two-way repeated measures ANOVA revealed an effect of cues (F(4,60)=16.6, *P*<0.05), no effect of days (F<1) and no interaction (F(16,240)=1.0). *Post hoc* analyses showed no differences between the auditory cues (all *q*<1, *P*>0.05), and a consistent difference between the visual cues (min *q*=9.1, *P*<0.05) across the conditioning days. (**c**) Compound training. A two-way repeated measures ANOVA on the compound probe phase (day 6) found a main effect of compound type (F(4,60)=20.6, *P*<0.05), a main effect of trial blocks (F(3,45)=6.4, *P*<0.05), and an interaction (F(12,180)=1.9, *P*<0.05). The Control differed from Omission but not from Overexpectation (see text). Discriminative responding seen to the visual cues (*q*=8.41, *P*<0.05). One-way ANOVA on responding during the compound training phase (days 7–9) revealed an effect of cue on each day (min F(4,60)=16.9, *P*<0.05), due to differences between the omission and control compounds (see text), and the two visual cues (min *q*=8.0, *P*<0.05). (**d**) Post-reward period of compound probe phase. A two-way repeated measures ANOVA confirms a main effect of compound cues (F(4,60)=12.1, *P*<0.05), a main effect of trials (F(3,45)=9.5, *P*<0.05), and an interaction (F(12,180)=4.2, *P*<0.05). Control differed from overexpectation early but not late in training (see text). Other comparisons were not of interest; (**e**) Test. A two-way repeated measures ANOVA on test revealed a main effect of cue (F(2,26)=5.0, *P*<0.05), no main effect of trials (F(2,39)=2.6, *P*=0.06), and no interaction (F(6, 78)<1, *P*=0.55). Overexpectation and Omission each differed from Control but not from each other (see text); (**a**–**e**) Error bars represent s.e.m. (**f**) Correlation between the difference in conditioned responding during the post-reward period between overexpectation and control and the difference in conditioned responding between overexpectation and control during test.

Conditioned responding to the cues during the pre-conditioning and conditioning phases developed as expected, with high levels of magazine entries to the reinforced cues but not the non-reinforced (visual) cue by the end of each phase (Fig. 2a,b).

### Compound and test phases

The critical phase of the experiment, the compound phase, followed conditioning and consisted of the following three conditions: overexpectation, omission and control. The first day of compound training, the compound probe phase, was of primary interest from both a behavioural and neural perspective. This probe session presented the greatest mismatch between real and expected outcomes for the overexpectation and for the omission compounds. Therefore, it provided the best opportunity to examine how such a mismatch, formally referred to as negative prediction error, altered behavioural and neural responding in the two extinction (overexpectation and omission) and the control compounds. It also offered insight into how the combined presentation of two previously conditioned cues summed to influence behavioural and neural responding in the overexpectation condition.

Behavioural responding to the reinforced compounds, that is, overexpectation and control, was similarly high across trials of the compound probe session (that is, day 6, Fig. 2c), Tukey's *post hoc* comparison test for each block of four trials (max *q*=1.3, *P*>0.05), and during the subsequent days of compound training (that is, days 7–9, Fig. 2c, max *q*=1.7, *P*>0.05). The high level of responding to the reinforced compounds was in stark contrast to that seen to the non-reinforced omission compound, which started high and similar to the control compound (*q*=2.6, *P*>0.05), but quickly declined across trials (Fig. 2c, across the remaining three blocks of four trials, min *q*=5.0, *P*<0.05) and remained low on subsequent compound training days (min *q*=5.5, *P*<0.05). Linear regression analyses supported these results: the omission compound (F(3,45)=13.1, *P*<0.05), but not the other two compounds (max F=2, *P*>0.05) showed a linear trend across trials. Our data confirm a well-established finding, namely that conditioned responding to previously reinforced stimuli declines when these stimuli are no longer reinforced^1^. Put another way, the reduction in behavioural responding to the omission compound during the compound probe day reflected the change in reward expectation, namely expectation of no reward, which was preserved across subsequent days.

Notably, the overexpectation compound resulted in a heightened expectation of reward. Although the rats did not increase responding during presentation of the overexpectation compound when levels were already high, they did respond more during (1 s) and some time after (30 s) food pellet delivery compared with the control compound (Fig. 2d), as if checking for additional reward. This difference in post-compound responding between the overexpectation and control conditions was present during the first half (Tukey's *post hoc* comparison test, *q*=3.9, *P*<0.05) but not during the second half (*q*=1.3, *P*>0.05) of the compound probe session, the latter result being consistent with updated expectations.

To verify that the omission and overexpectation training reduced the expectation of reward to the omission and overexpectation cues but not to the control cue, rats were tested following the end of the fourth (also last) day of compound training. The test consisted of the non-reinforced presentation of each of the three auditory cues intermixed with reinforced presentations of the previously trained visual cue. In line with predictions, Fig. 2e shows that responding on the test was similar between the two extinction cues (*q*=1.2, *P*>0.05) and lower compared with the control cue (overexpectation: *q*=3.1, *P*<0.05; omission: *q*=4.3, *P*<0.05). Notably, the overexpectation effect observed earlier during the post-reward period in the compound probe (Fig. 2d) predicted the low level of responding to the overexpectation cue on test (Fig. 2e). That is, there was a strong inverse correlation between the difference in number of magazine entries during the post-reward period following the presentation of the overexpectation and control compounds, and responding during the individual overexpectation and control cues on test (Fig. 2f; *r*^2^=0.62, *P*<0.05). In other words, the greater the overexpectation of reward during the first half of the compound probe, the lower the expectation of reward to the overexpectation cue on test 4 days later.

### CN neurons track reduction in reward expectancy

CN neurons did not signal the discrepancy between the expected and actual rewards in overexpectation and omission (that is, they did not signal negative reward prediction errors) during the compound probe phase (see Supplementary Note 2).

**Figure 3:**
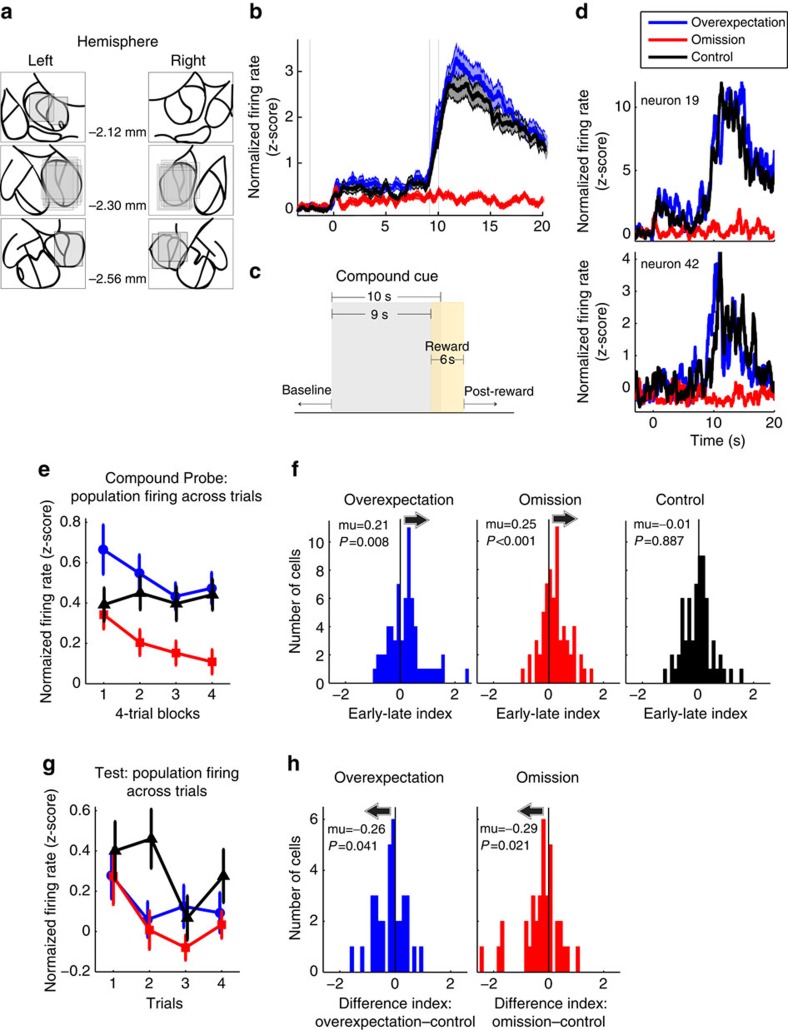
Neural firing of the reward-responsive population during the compound probe day. (**a**) Extent of recording sites within the CN, image adapted from ref. 43. Boxes represent the dorso-ventral path travelled by the electrodes and medio-lateral spread of the electrode bundle. (**b**–**d**) Onset of compound presentation indicated with 0 ms on *x*-axis. (**b**) Normalized neural firing of the reward-responsive population (65 cells) averaged across the 16 trials of the compound probe phase, shadings indicate the s.e.m. for each condition. (**c**) A graphical representation of the temporal resolution of a single trial. (**d**) Normalized neural firing across the 16 trials of the compound probe for two representative single neurons. (**e**) Normalized neural firing for the reward-responsive population during the 9 s of compound presentation across four-trial blocks during the compound probe session. Neural firing to the overexpectation compound is greater compared with the Control compound during the early but not late trials. Neural firing to the omission compound is similar to that of the control compound during the early trials but lower during the late trials. Error bars=s.e.m. (**f**) Distributions of difference indices (early-late trials) for overexpectation (blue, left panel), omission (red, middle panel) and control (black, right panel). The distributions were right shifted for the overexpectation and omission but not control compounds. (**g**) Neural firing for reward-responsive population to the 9 s of cue presentation across trials during test for each of the auditory cues. A two-way repeated measures analysis of variance revealed an effect of cue (F(2,70)=4.6, *P*=0.01), an effect of trials (F(3,105)=6.3, *P*<0.01) showing an overall decline in neural firing, but this was not dependent on cue as evidenced by a lack of interaction (F(6,210)=1.2, *P*=0.34). Neural firing was greater to the Control cue compared with the overexpectation and omission cues, *post hoc* comparison test reported in the main text. (**h**) Distribution of difference indices (from control) during the early trials (trials 1 and 2) for overexpectation (blue, left panel) and omission (red, right panel). Both distributions were shifted to the left.

Neural analyses were performed on the trials of the compound probe day, that is, the first day of compound training. In all, 87 units were recorded from the CN during this session (Fig. 3a), and 65 of those units were reward-responsive, that is, each cell showed an increase in firing during the 6 s reward period (1 s reward delivery+5 s post-reward delivery) compared with the 5 s pre-cue baseline (min t=2.4, *P*<0.03). Although this population of neurons fired to reward, it also fired during the first 9 s of the compound cues that preceded reward delivery reflecting reward expectancies. This is depicted on Fig. 3b alongside a graphical representation of the timeline of stimuli delivery (Fig. 3c) and two representative individual single units (Fig. 3d). In the 65 reward-responsive neurons recorded on the first day of compound training, reward expectancy was examined using the first 9 s of the compound presentation. A two-way repeated measures analysis of variance on compound evoked firing with compound and trials as factors confirmed a main effect of compound (Fig. 3e; F(2,128)=14.1, *P*<0.05), a main effect of trials (F(3,192)=6.6, *P*<0.05) and an interaction (F(6,384)=2.2, *P*<0.05). Although the expectation of reward to the omission and control compounds was similar at the start of the compound probe session, it diverged by the end of this session such that the omission compound signalled fewer or no rewards compared with the control compound. This was reflected in both the behavioural responses (see earlier) and neural responses. Compared with the control compound, neural firing in response to the omission compound was similar during the early trials (trials 1–4, Tukey's *post hoc* comparison test, *q*=1.3, *P*>0.05, Fig. 3e) but lower during the late trials (trials 13–16, *q*=6.5, *P*<0.05) of the compound probe day. When presenting two previously conditioned cues in compound, an overexpectation of reward was generated. In other words, more rewards were expected during the overexpectation compound compared with the control compound. Neural firing to the overexpectation compound reflected the following: compared with the control compound, neural firing to the overexpectation compound was greater during the early trials (Fig. 3e; *q*=3.9, *P*<0.05) and equivalent during the late trials (*q*<1) of the compound probe. A linear regression analysis confirmed the change in neural firing to the overexpectation and omission compounds, which showed a decline across trials (overexpectation: F(1,257)=3.8, *P*=0.05; omission: F(1,257)=8.0, *P*<0.05), but not for the control compound (F(1,257)=1.6, *P*>0.05). See Supplementary Note 2 and Supplementary Fig. 2 for neural firing during the conditioning phase and days 7–9 of the compound training.

These findings were also reflected at the level of individual neurons. We computed an index of change for each cell by taking the cue-evoked neural firing to each compound at the start (early trials: 1–4) minus that at the end (late trials: 13–16) of the compound probe session. We obtained a distribution of positive indices (Fig. 3f), reflecting a decrease in neural firing from the early to the late trials, for the overexpectation (μ=0.21, t(64)=2.7, *P*<0.05) and omission (μ=0.25, t(64)=4.1, *P*<0.05) compounds but not for the control compound (μ=0.03, t(64)=−0.1, *P*>0.05). In addition, the overexpectation and omission compound distributions each differed from the control compound distribution (overexpectation versus control: t(64)=2.5, *P*<0.05; omission versus control: t(64)=3.5, *P*<0.05). These results show that the reward-responsive population represented a somewhat homogeneous set of cells, which showed a decline in firing across trials to the overexpectation and omission compounds, but not the control compound.

We also examined changes in neural firing as a result of overexpectation and omission training in the test session after compound training. We recorded from 66 neurons in this session, 36 of which were reward-responsive during presentation of the reinforced visual stimulus (the only reinforced stimulus) on test. Neural firing in these neurons reflected the differences in reward expectancy associated with the individual auditory cues, such that the overexpectation (Tukey's *post hoc* comparison test, *q*=2.8, *P*<0.05) and omission cues (*q*=4.2, *P*<0.05) showed equivalent levels of firing to each other (*q*=1.4, *P*>0.05) but lower levels of firing compared with the control cue (Fig. 3g). Analyses at the level of the individual neuron confirmed this by showing a negative shift in the distribution of indices representing the difference in firing between the overexpectation and the control cues (μ=−0.26, t(35)=−2.1, *P*<0.05), and the omission and the control cues (μ=−0.29, t(35)=−2.4, *P*<0.05) during the initial trials of test (Fig. 3h). Taken together, these results confirm that on test, firing to the overexpectation and omission cues reflected the expectation of fewer or no rewards compared with the control cue.

### A common population subserves overexpectation and omission

**Figure 4:**
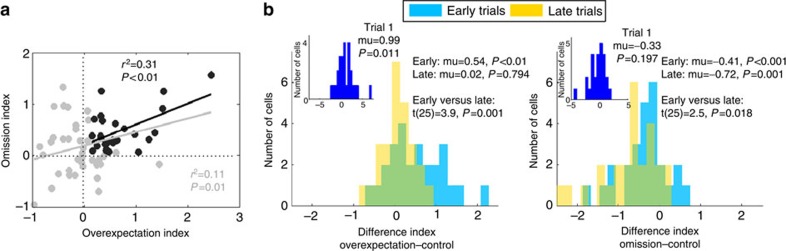
Neural firing of the common population of cells during the compound probe day. In all, 26 cells showed decline in neural firing across trials to both overexpectation and omission. This number of cells was greater than chance: a Monte Carlo simulation with one million repetitions reported that on average seven cells (full range: 0–22) were reward-responsive and showed a decline in neural firing to both the overexpectation and omission compounds from the early to the late trials (t(99999)=−7.7 × 10^3^, *P*<0.05). This was confirmed by a *χ*^2^-test of independence (*χ*^2^=13.5, *P*<0.05). (**a**) Correlation of the change in neural firing (early-late trials) between the overexpectation and omission compounds. This relationship was trial-specific and dependent on the true overexpectation–omission pairs. Randomly shuffling the trials in an independent manner disrupted this relationship: The true correlation was different to the correlation distribution obtained from a million permutations of the data (*P*=0.0103). Randomly shuffling the order of the neurons for Omission while maintaining the order of the neurons for overexpectation also disrupted the correlation. The correlation coefficient obtained by the true unshuffled data were different from the distribution of correlation coefficients obtained following one million randomized permutations of the data (*P*=0.0053). (**b**) Distribution of difference indices (from control) for overexpectation and omission during the first trial (insets), and during the early (blue bars) and late (yellow bars) trials of the compound probe. The distribution of these indices for the overexpectation compound was positively shifted during the early trials (*μ*=0.54, t(25)=4.2, *P*<0.05) with this shift being greatest on the very first trial (μ=0.99, t(25)=2.7, *P*<0.05, see inset), while it was centred on zero during the late trials (*μ*=0.02, t(25)=0.3 *P*>0.05). The shift in firing to the overexpectation compound relative to the control compound was significant (t(25)=3.9, *P*<0.05). The distribution of the indices for the omission compound was negatively shifted during the early trials (μ=−0.41, t(25)=−3.8, *P*<0.05) and even more during the late trials (*μ*=−0.72, t(25)=−5.5, *P*<0.05, with a significant difference between the two distributions (t(103)=2.5, *P*<0.05). The difference in firing between the omission and control compounds on the first trial was centred on zero (μ=−0.33, t(25)=−1.3, *P*>0.05, see inset).

A common teaching signal, namely negative prediction error^4^, regulates the behavioural (and neural) changes seen to the overexpectation and omission compounds. However, whether that learning is apportioned to a common population of neurons or maintained separately is an open question. To explore this, we compared changes in firing with the changes in expectancies to these cues within each neuron. We found that 26 cells from the population of reward-responsive cells (*n*=65) recorded on the compound probe day (reported above) showed a decline in neural firing to both the overexpectation and omission compounds (min t(898)=2.0, *P*<0.0455), a number greater than expected by chance (Fig. 4 legend for statistics). Further, the decline in neural firing from the early to the late trials in the overexpectation compound correlated with the same decline in the omission compound (Fig. 4a; *r*^2^=0.31, *P*<0.05), but neither of which correlated with the control compound (overexpectation: *r*^2^=0.03, *P*=0.41; Omission: *r*^2^=0.00, *P*=0.83). Shuffling among the 16 trials (Fig. 4a legend for statistics) confirmed that the relationship between firing to the overexpectation and omission compounds was trial-specific, that is, it depended on the true sequence of the trials. Shuffling between neurons to disrupt the neuron correspondence between overexpectation and omission (for example, cell X for overexpectation matched to cell Y for omission) also disrupted the relationship. This shows that the correlation in firing rate change across time between the overexpectation and omission compounds was not spurious, was specific to the true order of the trials and was not due to the inherent firing rates of the neurons.

To summarize, the cells in this common population signalled the decrease in expected reward in the overexpectation and omission conditions in a correlated manner. This change in neural firing could represent an increase in attention driven by prediction error^14^, or the updating of the reward expectancies. These two possibilities make different predictions as to how neural activity to the two extinction compounds would compare with that of the control compound across trials. If this neural change reflected changes in attentional processing, then firing during the overexpectation and omission compounds would be greater than that to the control compound during early but not late trials, since in each case prediction errors should drive similar increases in attention in the early trials. In contrast, if updated reward expectancies drove this change (as was the case for the entire reward-responsive population) then firing during the overexpectation compound should be greater than the control compound during the early but not late trials, whereas firing during the omission compound should be lower than the control compound during the late trials but not during the early trials.

To explore this question, we examined an index of the difference in firing between the overexpectation (Fig. 4b, statistics in legend) or omission (Fig. 4c, statistics in legend) versus the control compounds. The distribution of these indices during the first trial, the early trials and the late trials confirm that compared with the control compound, firing to the overexpectation compound was greater at the start but equivalent by the end of compound training, firing to the omission compound was equivalent during the first trial with a gradual difference emerging across subsequent trials, with the greatest difference presenting during the late trials. These analyses show that this common population of cells tracked reward expectancy, and did not reflect an attentional process.

### Neural firing in the CN predicts behavioural changes

**Figure 5:**
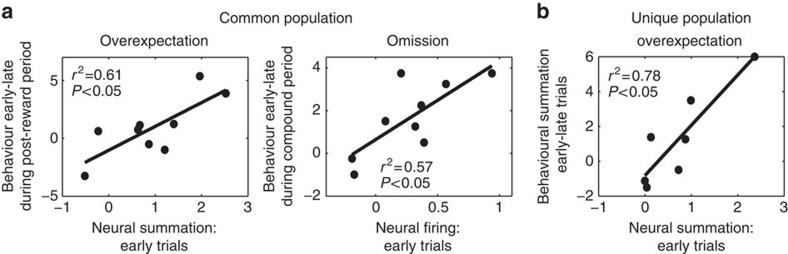
Relationship between neural firing and behaviour. (**a**) Significant positive correlation between neural summation and decline in behavioural summation for overexpectation in the common population (left panel); significant positive correlation between neural firing to the omission compound in the common population during the early trials and decline in behavioural responding (right panel); (**b**) significant positive correlation between neural summation and behavioural summation for the overexpectation-unique population.

Given that the behavioural and neural responding reported here are in line with theoretical stipulations of learning^5^, it would be important to examine whether neural firing in the common population predicts changes in behavioural responding. Error-correcting mechanisms of learning predict that the greater the expectation of reward, then the greater the negative prediction error that would be generated when fewer or no rewards are delivered, and thus the greater the decline in the subsequent behavioural response. In the overexpectation condition, the difference in neural firing between the overexpectation and control compounds during the early trials of the reward period (see Supplementary Note 3 and Supplementary Fig. 3) reflected the inferred expectation of additional pellets, that is, neural summation. This difference in neural firing predicted the reduction across time in behavioural responding during the post-reward period following the overexpectation compound (Fig. 5a left panel; *r*^2^=0.61, *P*<0.05). The regression coefficient confirmed this relationship (*β*=2.06, confidence interval (0.59, 3.53)). Similarly, neural firing during the early trials of the Omission compound reflected the greatest expectation of reward for the omission condition, and this neural firing predicted the decline in behavioural responding across time during the omission compound (Fig. 5a right panel; *r*^2^=0.57, *P*<0.05), also confirmed by the regression coefficient (*β*=3.68, confidence interval (0.84, 6.52)). Critically, the common population of cells uniquely predicted the relationship between neural firing and behaviour. The population of cells (see Supplementary Note 4 and Supplementary Fig. 4) that uniquely track the decline in firing to overexpectation (and not omission; *n*=11 cells, *r*^2^=0.38, *P*=0.14, *β*=1.64, confidence interval (−0.78, 4.05) see Supplementary Figure 5a) or omission (and not overexpectation, *n*=16 cells, *r*^2^=0.03, *P*=0.66, *β*=0.54, confidence interval (−2.24, 3.31) see Supplementary Fig. 5a) did not show a relationship between neural firing and changes in behaviour.

Although neural summation at time of the reward period in overexpectation-unique population did not predict the decline in behaviour, it predicted behavioural summation (Fig. 5b; *r*^2^=0.78, *P*<0.05, *β*=2.89, confidence interval (1.14, 4.65)). This was not the case for the common population: neural summation did not predict behavioural summation (*r*^2^=0.36, *P*=0.09, *β*=1.28, confidence interval (−2.53, 1.52) see Supplementary Fig. 5b). To elaborate, neural summation in the overexpectation-unique population seems to specifically correlate with the higher level of responding that results from reward overexpectation, but not the decline in responding that ensues when the expectation of double reward is met with a single one.

The differences in the correlations between neural firing and behaviour across the different populations reported above must be treated with caution due to the low number of neurons used to derive the correlations, particularly in the case of the unique populations. Indeed, in the case of Overexpectation, summation is important for the reduction in outcome expectancy, thus even if two distinct populations regulate behavioural summation versus behavioural change, these populations must interact and influence their firing, be it in a uni or bidirectional manner.

## Discussion

The ability to update expectations about environmental events is key to adaptive behaviour and survival. Using two distinct paradigms that result in extinction learning, namely simple extinction (termed omission here) and overexpectation, we show that CN cells represent expected outcomes resulting from explicitly trained or flexible integration of associative relationships, update outcome expectation real-time as a result of changing contingencies, and do so in a correlated manner between the two paradigms. As a result these findings extend prior work using temporary or permanent cell-silencing techniques that have implicated the CN in learning as a result of outcome omission^8^,^15^ and overexpectation^9^. Further, our recording data distinguish between prediction error signalling versus associative updating, which ensue when delivered outcomes fall short of meeting expectations. The present data provide insight into CN function real-time during this learning.

Before delving into the functional role of the CN in simple extinction (omission) and overexpectation as revealed by the present data, a number of important alternate accounts for the increase in cell firing to the overexpectation compound can be excluded. The presentation of an auditory and visual compound during the compound phase presents both a novel as well as a multisensory stimulus. Critically, the novelty, multisensory and integrative nature of the overexpectation compound matched those in the omission and control compounds, thus making these constructs an unlikely explanation for the higher level of cell firing in overexpectation compared with the other two compounds. In addition, the behavioural and neural data obtained from the overexpectation compound do not represent greater certainty of the arrival of two pellets following overexpectation compared with the other two compounds. If so, then this certainty would be matched by the delivered outcome and should not result in a lower level of responding on test to the overexpectation cue compared with the control cue.

Of course, brain regions other than the CN have been shown to be involved in learning under conditions of reward omission or overexpectation. Areas upstream such as the orbitofrontal cortex (OFC) and the basolateral amygdala (ABL) have been implicated in these processes. For example, cell firing in both OFC and ABL tends to track learning, increasing when cues are paired with reward and decreasing when they are unrewarded^16^,^17^,^18^,^19^,^20^,^21^. More specific to the current study, cells in both areas show summation of neural responses and reduction in neural firing to the target cue as a result of overexpectation training^17^,^20^. The neural summation observed in ABL was abolished by OFC lesions, whereas simple associative changes in firing were unaffected^20^. Interestingly, the respective roles for the OFC and ABL in learning from fewer-than-expected outcomes also diverge when examined causally; disruption of OFC function via GABA agonists or optogenetic means impaired learning from overexpectation^9^,^17^,^22^, but not learning from reward omission, whereas ABL inactivation left overexpectation intact^14^,^22^. These somewhat conflicting findings further exemplify the importance of studying both paradigms in parallel. The present study examining the processing of both types of extinction in single units within CN suggests that this structure serves as a site of convergence for information about fewer-than-expected outcomes generally in a task-independent manner.

The finding that a common population of CN cells processes the decline in reward expectancy in omission and overexpectation supports the idea that the CN is a site for convergence of information when fewer-than-expected rewards are delivered at both a structural and single-unit level. Notably, changes in neural firing in this common population across learning in omission predicted similar changes in overexpectation. Thus, despite the large procedural differences between the two behavioural paradigms, cells in the CN reflected associative updating in those paradigms in a coordinated manner. It must be noted that since the omission and overexpectation compounds did not signal the same outcome (that is, one predicted some pellets, although fewer-than-expected, while the other predicted none), the correlation in the change in neural firing across time cannot reflect the absolute value of outcome expectancy given the compounds, but instead some other information. That information is likely to be the reduction in the reward expectancy suffered by both compounds. If this reduction represents inhibitory learning, then it must be specific to cues that suffer loss in previously established associations, as recent work shows that the CN is not necessary when a novel cue is established as a conditioned inhibitor, that is, a signal for the omission of an outcome^10^. Our finding represents an important step forward in marrying learning theory and neuroscience; error-correcting theories account for omission and overexpectation in an identical manner^5^, and both paradigms have been shown to be subject to the disruptive effects of the passage of time^2^ and context change^3^. The present results contribute further support for the commonality of omission and overexpectation at the cell level.

In addition, the present data provide another important insight that it is the common population (not the unique populations) of cells that track the decline in reward expectation and behaviour. In other words, both learning and changes in behaviour that result from omission of an expected reward or the inflation of an expected reward expectancy have a common neural locus. This carries the implication that disruption in one of these processes, such as overexpectation, may be predictive of a general inability to learn from situations when fewer-than-expected rewards are delivered, such as in omission. Indeed work on addiction is suggestive of this; rats with history of exposure to cocaine show disruption in learning from overexpectation^23^, as well as resistance to omission (extinction) learning^24^.

It is not to say of course that neural (and behavioural^25^) processing under conditions of omission is identical to that of overexpectation. Indeed, the omission- and overexpectation-unique populations may serve to process other aspects of learning and behaviour. As suggested here, although neural summation in the common but not unique, populations predicted the decline in behaviour, neural summation in the overexpectation-unique, but not common population, predicted behavioural summation. Perhaps, the role of the overexpectation-unique population in processing summation carries implications that are unique in regulating the flexible integration of information across various sources, which are not necessarily easily altered in the face of violated contingencies. In other words, such cells could hold the key to understanding why individuals can be so resistant to updating their own false beliefs.

An alternative possibility for CN function in associative learning as driven by omission of expected outcomes is attention. Permanent and temporary lesion work done by Holland and colleagues^15^,^26^,^27^,^28^ have uncovered that cells in the CN are critical in regulating associative learning by modulating attentional mechanisms. Indeed, both omission and overexpectation present an opportunity for attention to be upregulated as a result of violation in outcome expectancies^29^ and the CN has been ascribed a role in regulating this attention specifically to cues that contribute to such violations and not to novel cues^10^. Although the correlated change in activity in both paradigms is suggestive of such an attentional process that modulates learning in both cases, the firing rate indicates that the information carried by the neural activity is related to outcome expectancy. A clear prediction that emerges of attentional accounts of learning is that firing to the overexpectation and omission compounds should be similar to each other but different from firing to the control compound. Our data did not show this pattern (see also Supplementary Note 5). Instead, the information carried by CN cells is consistent with with the idea that the CN processes general affective information with regard to outcome expectancy as revealed by Pavlovian-to-Instrumental transfer tests, which is in contrast to sensory-specific outcome information processed by the ABL^30^,^31^,^32^,^33^. Indeed, CN firing tracked the incentive value of each compound, which remained steady for the control compound as it did not suffer any violation in outcome expectancy, but declined to both the overexpectation and omission compounds. If the CN processes affective (as opposed to associative) information, then our results also suggest that the affective properties of outcomes can be integrated from various sources and become represented as a summed motivational component. This is particularly interesting as it allows for the possibility of different outcomes to be integrated in terms of their incentive value in the CN as a form of a common neural currency, while perhaps maintaining differential processing in other areas such as the ABL. The present results also show that losses in incentive value are processed in a correlated manner irrespective of the conditions under which they are achieved.

While the current study focuses on reward learning, the role of the CN in the aversive (fear) setting cannot be ignored. Indeed, the delivery of fewer-than-expected rewards in simple extinction (omission) and overexpectation can be said to activate an aversive motivational state^34^, thus allowing for the present set of results to be consistent with previous research implicating the amygdala in the regulation of emotional memories^35^ and particularly aversive or fear-eliciting events^36^. It must be noted, however, that in the fear setting a distinction between the lateral and medial subcomponents of the CN has emerged with the medial CN serving as an output relay to downstream structures and the lateral CN as the main input site involved in regulating the expression of learning^12^,^37^,^38^,^39^. In the reward setting, similar distinction has not been made, but studies show more fos expression in the medial compared with the lateral subregion of the CN under conditions of reward omission^40^,^41^ (but also see ref. 42), perhaps suggesting that learning in this context may be regulated in the medial CN. Future investigations of the role of the lateral and medial CN are required to better understand the intra CN dynamics in reward. In any case, our results taken together with many other investigations show that the role of the CN in learning and memory envelops not only explicit aversive events, such as fear and anxiety, but also aversive emotions that result from obtaining fewer-than-expected rewarding events^7^,^8^.

## Methods

### Subjects

Twelve naive male Lister Hooded rats were used in this experiment weighing between 343 and 425 g. Rats were obtained from Charles River Laboratories (Wilmington, MA, USA). All experimental procedures were in accordance with University of Maryland and National Institute of Drug Abuse guidelines for animal testing.

### Surgery and histology

Immediately before and during surgery, anaesthesia was induced and maintained using an isoflurane and oxygen mix. Rats were placed in a stereotaxic apparatus (Kopf Instruments) and burr holes were drilled in the skull directly above the area of implantation. Two sixteen-channel bundles of chronic drivable electrodes were placed bilaterally at 2.3 mm posterior and 4 mm laterally to bregma. The electrodes were lowered 7 mm below the surface of the brain at the site of implantation. Rats were allowed minimum 14 days (14–20 days) for recovery from surgery before the start of the behavioural and recording procedures.

### Electrodes

Electrodes were built in-house using formvar-insulated nichrome wire (80% nickel and 20% chromium; A-M systems, Sequim, WA, USA). The day before surgery, the bundle of wires were freshly cut to extend ∼1 mm below the cannula holding them together and were electroplated with platinum (H2PtCi6, Aldrich, Milwaukee, WI, USA) at an impedance of 300 K Ohms.

### Apparatus

The behavioural apparatus, where behavioural training and recording took place, consisted of aluminium chambers of black sloping walls narrowing to a 12 × 12 in floor area. The chamber floor consisted of a black plastic over a wire mesh, under which was an aluminium tray. The chamber was equipped with a recessed food cup at the center of the left sidewall, as well as two speakers, a mechanical clicker and two visual stimuli (Coulbourn Instruments) located on the same wall. A pellet dispenser was mounted on the outside of the wall and attached to the food cup, where the pellets were delivered. Head entries into the food cup were monitored by a photobeam.

### Stimuli

Three auditory and two visual stimuli were used in this experiment. The auditory cues were a 75 dB tone, 72 dB white noise and a mechanical clicker pulsing at a rate of 4 Hz (Coulbourn Instruments). The visual cues included one flashing light (light pulsing at a rate of 4 Hz) and one steady light. All cues lasted 10 s. The reward consisted of two grape-flavoured sucrose pellets.

### Procedures

Rats were food deprived to 85% of their body weight before the start of behavioural training. Before surgery, all rats received magazine training and 5 days of pre-conditioning. Magazine training consisted of placement into the behavioural chambers with 20 sucrose pellets made available in the magazine. The session lasted 30 min and two sucrose pellets were delivered with an average intertrial interval (ITI) of 4 min (2.5–5.5 min). During the subsequent 4 days, all rats received pairings between each of three auditory cues (clicker, white noise and tone—counterbalanced) with two sucrose pellets as well as discriminative conditioning with two visual cues (steady light and a flashing light—counterbalanced), one of which was reinforced with two sucrose pellets, while the other was not. Sucrose pellet delivery occurred at the 9th second of cue presentation and took a second to complete. The average ITI during Pavlovian training was 90 s (60–120 s). The purpose of this pre-training was to ensure that all rats were able to perform in the task and were able to acquire the visual discrimination. Once rats had recovered from surgery, a conditioning phase commenced and lasted 5 days. Training was identical to that described for the pre-surgery period. On day 6, compound training began which consisted of the presentation of each of the auditory cues in compound with a visual cue. For the overexpectation condition, one of the auditory cues (for example, tone) was presented simultaneously with the previously reinforced visual cue (for example, flashing light). For the omission condition, one of the remaining auditory cues (for example, white noise) was presented in compound with the previously non-reinforced visual cue (for example, steady light). For the control condition, the third auditory cue (for example, clicker) was presented together with the previously non-reinforced light (for example, steady light). The role of each auditory cue and each visual cue was counterbalanced. Both the overexpectation and control compounds were each reinforced with two sucrose pellets in a manner identical to that described earlier whereas the omission compound was not reinforced. Compound training consisted of a single 2 h session comprised of 16 presentations of each of the 3 compounds, 16 reinforced presentations of the visual cue that was previously paired with sucrose pellets and 16 non-reinforced presentations of the visual cues that was not previously paired with reward. For the compound training phase, the overexpectation compound was intermixed with the reinforced visual cue in blocks of 32 trials, while the omission and control compounds were both intermixed with the non-reinforced visual cue in two blocks of 24 trials each. These blocks of training were delivered sequentially while the rats remained in the chambers. Stimuli duration, reward delivery and the ITIs were identical to that of conditioning. Compound training lasted 4 days. To confirm that compound training was effective, a test was performed immediately following the last training session on day 4. This test consisted of four non-reinforced presentations of each of the auditory cues among pairings of the previously reinforced visual cue with sucrose pellets. All behavioural training occurred in the same context as that used for magazine training. Behavioural data were recorded by Coulbourn Instruments software and analysed with Matlab.

### Neural analyses

Analogue signals of the neural data were collected using several identical Plexon MAP systems and sorted online using Sort Client. The neural signal was amplified, filtered and waveforms with 2.5:1 signal-to-noise ratio showing stable activity (no drifting or disappearance of waveforms across the session on the PCA scatter plot with time as factor) were selected for subsequent analyses. The units were subsequently verified using a template-matching algorithm through Offline Sorter, Plexon Inc., and only units that showed stable firing across the day's behavioural session were used for further analyses. Unit time stamps and event markers were extracted with Neuroexplorer, verified and further analysed with Matlab. Time bins of 20 ms were constructed and a rectangular smoothing function using a 100 ms window was applied to the neural data across the duration of each training session. Neural data were normalized on a trial-by-trial basis to obtain *z*-scores relative to baseline firing. The mean and standard deviation of the *z*-score normalization were taken from the first 5 s of the pre-CS period (10 s long) for the corresponding trial and cue.

Subsequently, cells were screened for excitatory reward responses. Reward responses were defined as an increase in neural firing during the 6 s reward period (1 s of reward delivery plus an additional 5 s post-reward period), from the 5 s pre-compound baseline using a Student's *t*-test. This additional and prolonged period was necessary due to the nature of the reward (pellets), allowing for delivery, obtaining and consuming the food reward. Subsequent analysis of variance, Tukey's *post hoc* comparison tests or Student's *t*-tests were used to further examine the change in neural firing of these reward-responsive cells. These analyses focused on the reward expectation period, that is, first 9 s of compound cue. The reward expectation period was defined as the first 9 s of the 10 s cue per compound presentation. The last second of the cue per compound was not included in the reward expectation analyses period as it consisted of reward delivery.

### Data availability

Programming code and data reported in this manuscript are available on request from the authors.

## Additional information

**How to cite this article:** Iordanova, M. D. *et al.* Neural correlates of two different types of extinction learning in the amygdala central nucleus. *Nat. Commun.* 7:12330 doi: 10.1038/ncomms12330 (2016).

## Supplementary Material

Supplementary Information Supplementary Figures 1-5 and Supplementary Notes 1-5
