# Supplementary material for: Neural correlates of two different types of extinction learning in the amygdala central nucleus
Source: Nat Commun. 2016 Aug 17;7:12330. doi: 10.1038/ncomms12330 (PMC4992052; doi:10.1038/ncomms12330)
Supplement: Supplementary Information — Supplementary Figures 1-5 and Supplementary Notes 1-5 [file ncomms12330-s1.pdf]

SUPPLEMENTARY FIGURE 1

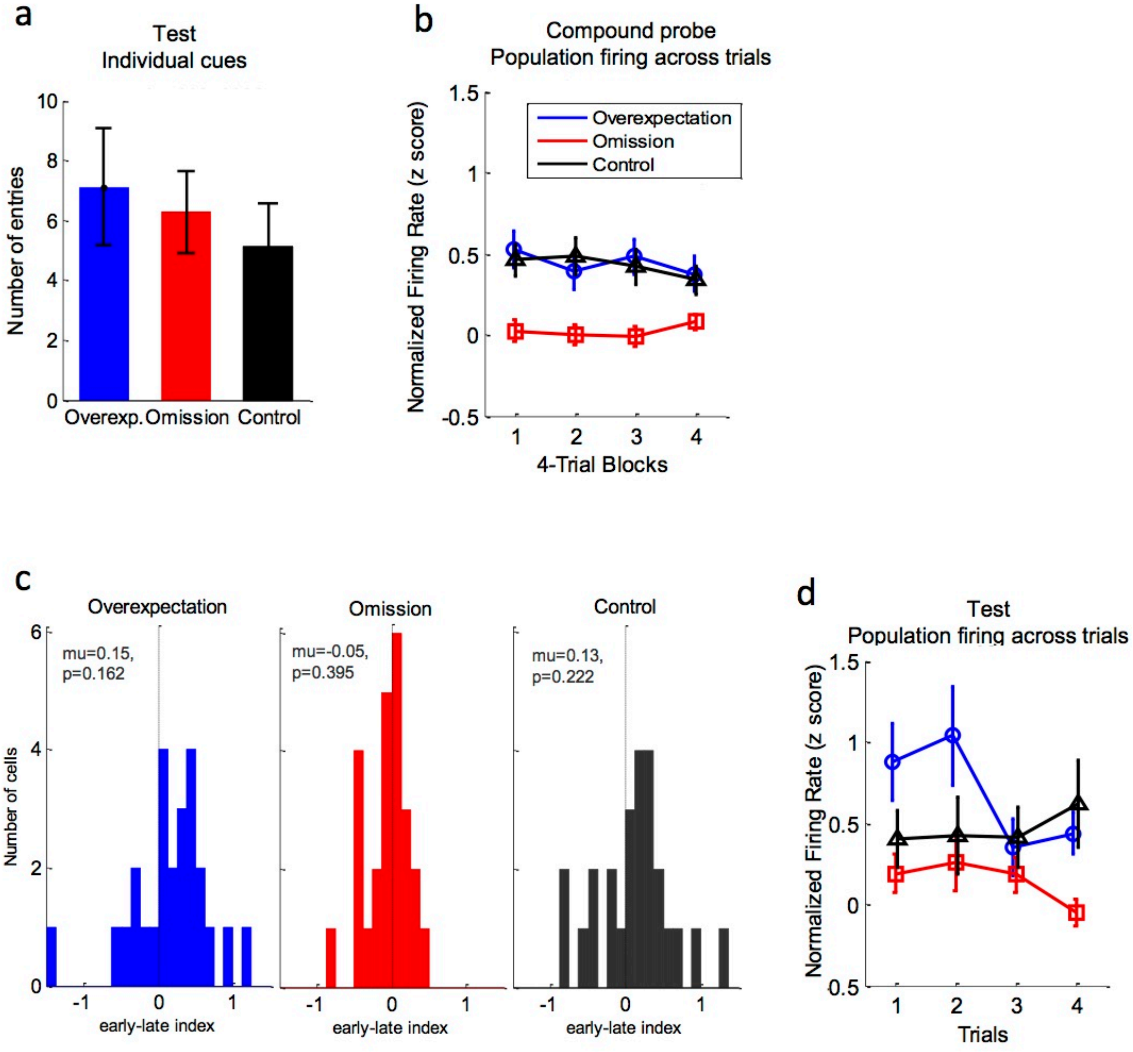

Supplementary Figure 1. Neural firing in the absence of behavioral Overexpectation or Omission. a. Behavioral responding (number of magazine entries) during Test. No differences were obtained between the three auditory cues on Test; **b.** Normalized neural firing during the compounds. No differences in neural firing were obtained between the Overexpectation and Control compounds, whereas firing to the Omission compound was consistently lower compared to Control compound; **c.** Distribution of difference indices (early-late) for each of the three compounds (Overexpectation, left panel; Omission, middle panel; Control, right panel). All distributions were centered on zero. **d.** Normalized neural firing to each of the auditory cues on Test. No differences in neural firing were obtained between the cues.

SUPPLEMENTARY FIGURE 2

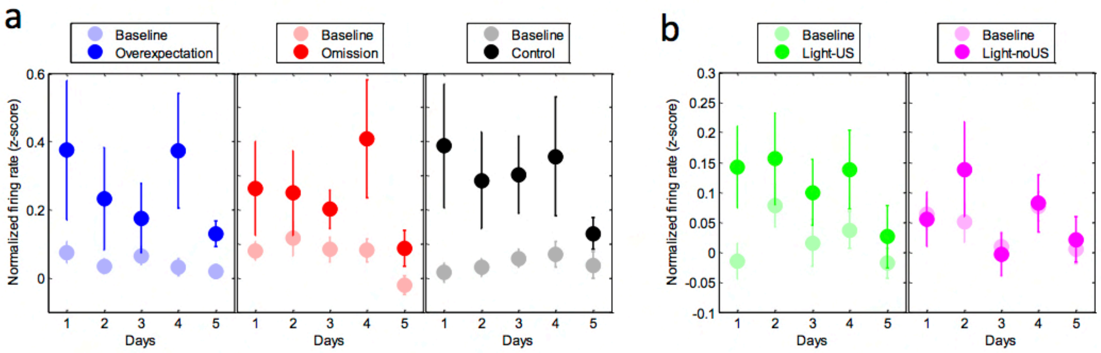

Supplementary Figure 2. Neural firing of the reward-responsive population during the Conditioning phase. a. Normalized neural firing of the reward-responsive population for each of the compounds compared to their corresponding baseline (Overexpectation - blue, baseline – light blue, left panel; Omission - red, baseline – light red, middle panel; Control - black, baseline – grey, right panel) for each day of the Conditioning phase. Error bars = S.E.M.; **b.** Normalized neural firing of the reward-responsive population for each of the reinforced visual cue (Light-US) and non-reinforced visual cue (Light-noUS) compared to their corresponding baseline (Light-US - green, baseline – light green, left panel; Light-noUS – pink, baseline – light pink, right panel) for each day of the Conditioning phase. Error bars = S.E.M.

SUPPLEMENTARY FIGURE 3

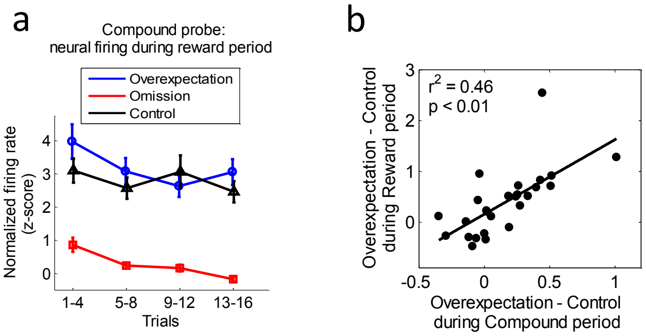

Supplementary Figure 3. Neural firing at time of reward for the common population. a. Normalized neural firing of the common population during the post-reward period. Firing was greater when reward was delivered (following Overexpectation, blue, and Control, black) compared to when no reward was delivered (Omission, red). Firing following the Overexpectation compound is greater compared to following the Control compound during the early trials. **b.** Correlation of neural summation (Overexpectation – Control during the early trials) between the compounds and post-reward periods in the common population.

SUPPLEMENTARY FIGURE 4.

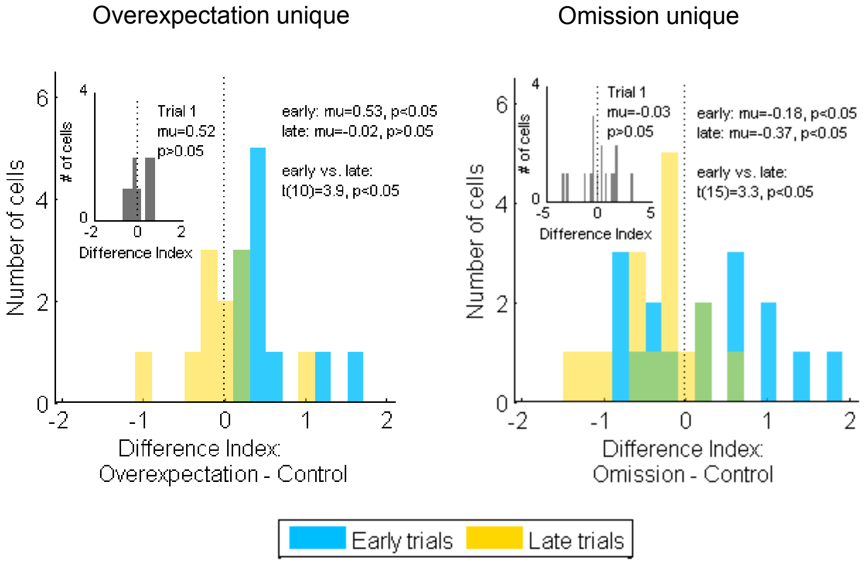

Supplementary Figure 4. Neural firing to the unique populations. Distribution of difference indices (from Control) for the Overexpectation (left panel) and Omission (right panel) unique populations. Distributions are shifted to the right for the Overexpectation unique population for the early but not late trials. Distributions are shifted to the left for the Omission unique population for the late but not early trials.

SUPPLEMENTARY FIGURE 5.

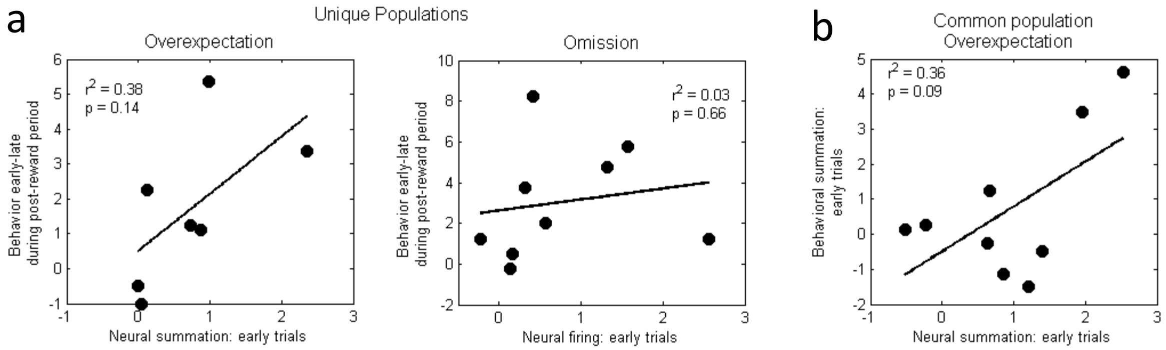

Supplementary Figure 5. Additional correlations in unique and common populations. a. Correlation between neural summation and decline in behavioral summation for the Overexpectation unique population is not significant (left panel); Correlation between neural firing to the Omission unique compound in the common population during the early trials and decline in behavioral responding is not significant (right panel); **b.** Correlation between neural summation and behavioral summation for Overexpectation in the common population shows a tend but is not significant.

SUPPLEMENTARY NOTE 1. Analyses of failed Overexpectation and Omission learning.

Sessions categorized as exhibiting poor performance were based on behavioral data obtained from the Test, which showed no Overexpectation or Omission effects (see below). The behavioral and corresponding neural data from these sessions were analyzed separately from the data in the main text. Analyses of the neural data from these failed sessions allow for further determining whether the changes in neural firing reported in the main text are only present in sessions that show evidence of Overexpectation and Omission training on Test. If so, then the pattern of neural firing obtained from the failed sessions should not mirror that reported in the main text. To examine this prediction, data from these eight failed sessions were analyzed during the Compound Probe and Test phases. The results confirmed that the changes in neural firing reported in the main text were unique to sessions that resulted in behavioral evidence of Overexpectation and Omission on Test.

Six rats across eight sessions failed to show behavioral evidence of Overexpectation or Omission training (Supplementary Figure 1a): There were no differences in conditioned responding on Test across cues for these rats. An ANOVA on Test revealed no effect of cue ($F(2,14)=3.15$, $p>0.05$), an effect of trials ($F(2,21)=3.78$, $p<0.05$) showing a decline in responding across Test, and no interaction ($F<1$). In addition, to poor performance on Test, these rats failed to show Overexpectation during the Compound Probe session as evidenced by a lack of behavioral summation: Magazine entries during the post-reward period were identical following the presentation of the Overexpectation and Control compounds. An ANOVA revealed no effect of compound ($F(2,14)=3.5$, $p>0.05$), an effect of trials ($F(3,21)=23.4$, $p<0.05$), showing a decline in conditioned responding, and no interaction ($F(6,42)=1.8$, $p>0.05$). In other words, the higher level of behavioral responding following the presentation of the Overexpectation compared to the Control compound reported in the main text was not present during the same sessions of the rats that failed to show evidence of Overexpectation and Omission learning on Test.

Thirty-two neurons were recorded from on Compound Probe day and 25 of those neurons were found to be reward responsive. Critically, neural firing at time of reward expectation i.e. during the compounds revealed a different pattern of firing to that reported in the main body of the manuscript: There was no evidence that these cells tracked any changes in associative relationships. An ANOVA of the cue-evoked firing confirmed a main effect of compound (Supplementary Figure 1b; $F(2,48) = 16.36$, $p < 0.05$), no effect of trials ($F(3,72)<1$, and no interaction ($F(6,144)=1.1$, $p>0.05$). Post-hoc Tukey analyses of the effect of cues revealed no differences in firing between the Overexpectation and Control compounds (each $q<1$, $p>0.05$) during the Compound Probe session, whereas firing to the Omission compound was different to the Control compound (min $q=3.29$, $p<0.05$) from the onset of this session. The lack of change across time were confirmed by a lack of linear trends for each of the compounds (max $F(1,385)=1.5$, $p>0.05$). Individual neuron analyses also confirmed no change across trials for any of the three compounds. Change indices representing firing during the early trials minus firing during the late trials were not different from zero for any of the compounds (Supplementary Figure 1c, max $t(17)=-1.5$, $p>0.05$). Finally, as neural firing did not reflect any change in associative relationships across the Compound Probe, no corresponding differences between the cues were seen on Test (see Supplementary Figure 1d). An ANOVA revealed an effect of cue $F(2,38)=8.8$, $p<0.05$), no effect of trials $F(3,57)=1.9$, $p>0.05$) and no interaction $F(6,144)=2.0$, $p>0.05$). To further examine the effect of cue, three paired-samples t-tests using Bonferonni correction revealed no difference between the Overexpectation and Control cues ($t(38)<1$, $p>0.05$) nor between the Omission and Control cues ($t(38)=-1.7$, $p>0.05$), but a difference between the Overexpectation and Omission cues ($t(38)=2.9$, $p<0.05$).

SUPPLEMENTARY NOTE 2. Additional Neural analyses of Overexpectation and Omission.

*CN neurons do not signal negative reward prediction errors***.** In order to determine whether CN neurons signal negative reward prediction errors, neural firing at time of reward was compared following

presentation of the Overexpectation and Omission compound compared to the Control compound in each of the 87 cells recorded from during the Compound Probe day. It was expected that neurons signaling negative reward prediction errors should show a change (decrease or increase) in firing when no (Omission) or fewer (Overexpectation) than expected rewards were delivered compared to no prediction error conditioning (Control). Greater firing at time of reward following Control compared to Overexpectation (t-test, $p<0.05$) was obtained in only four cells, a number significantly lower than chance ($t(999)=185.5$, $p<0.05$). Only one cell not part of the reward responsive population characterized below (and shown not to reflect negative prediction error) showed greater firing following Overexpectation compared to Control (t-test, $p<0.05$). In the case of Omission, there were no neurons that showed lower firing rates compared to Control that were not part of the reward responsive population that characterized below (and shown not to be signaling negative reward prediction errors). Nine cells showed greater firing following the Omission compared to the Control compound, but this number was below chance ($t(999)=139.2$, $p<0.05$). These results indicate that CN cells in the present study did not signal negative reward prediction errors induced either by overexpectation or omission of reward.

Conditioning phase. The high level of behavioral responding to the auditory cues seen across the five days of Conditioning reflects the expectation of reward during the presentation of the cues. We sought to determine whether changes in neural firing in the CN similarly reflected the expectation of reward upon presentation of the trained cues. During Conditioning, single-cell activity was recorded from a total of 244 neurons (Day 1: 41; Day 2: 47; Day 3: 49; Day 4: 53; Day 5: 54) from all rats and 116 of those units (Day 1: 21, Day 2: 25, Day 3: 22, Day 4: 25, Day 5: 23) were found to be reward-responsive. Such cells were defined as having shown an increase in their firing from baseline (5 second period prior to cue presentation) during the reward period (6 second commencing at time of reward delivery), as confirmed by a paired-samples t-test across the 16 trials (min $t(15)=2.1$, $p=0.049$). In order to determine whether these neurons signaled reward expectation upon presentation of the cues, neural firing was examined during the

cue presentation period prior to reward delivery. Supplementary Figure 2a shows that a greater change in neural firing was obtained during the presentation of the auditory cues compared to the baseline period for each day during the Conditioning phase (Day1: min $t(15)=5.0$, $p<0.05$; Day2: min $t(15)=3.9$, $p<0.05$; Day3: min $t(15)=3.4$, $p<0.05$; Day4: min $t(15)=4.9$, $p<0.05$; Day5: min $t(15)=4.5$, $p<0.05$), yet no differences were obtained between the cues on any day (max $F(2,42)=1.56$, $p\geq0.05$). Similarly, a consistent difference in neural firing during the reinforced visual cue compared to baseline was present during Conditioning for all days except one (Days 1-4: min $t(15=2.2$, $p<0.05$; except Day 5: $t(15)=1.1$, $p=0.29$, Supplementary Figure 2b). The opposite held true for the visual cue that was not reinforced, namely the change in neural firing during the cue was no different to that exhibited during the baseline (Days 1, 3, 4, 5: each $t(15)<1$, $p>0.52$; Day 2: $t(15)=4.1$, $p<0.05$).

Neural firing during Compound training: Days 7-9. No spontaneous recovery in neural firing was seen at the start of Day 2 of Compound training: Neural firing remained similar to the Overexpectation compound (vs. Control, $F(2,160)=2.60$, $p>0.05$) and lower to the Omission compound (vs. Control, $F(2,160)=4.82$, $p<0.05$). Across subsequent Compound training days, neural firing to the compounds remained stable: Overexpectation and Control compounds remained equivalent (each $F<1$), and the difference in firing seen between the Omission and Control compounds remained constant ($F(1,249)=3.7$, $p>0.05$).

SUPPLEMENTARY NOTE 3. Neural Firing to reward: Common population

Neural responses to reward during the Compound Probe in the common population exhibited a mixed profile that tracked reward delivery as well as aspects of reward expectation. Neural firing (averaged across all 16 trials) during the reward period was greater compared to the compound period ($t(25)=8.3$, $p<0.05$), and firing was greater to reward compared to baseline for the two conditions that received pellets i.e. Control ($t(25)=7.6$, $p<0.05$; Supplementary Figure 3a), and Overexpectation ($t(25)=8.2$, $p<0.05$). Greater

firing during the reward compared to the baseline period was also obtained for the condition that received no pellets, i.e. Omission ($t(25)=3.6$, $p<0.05$; Supplementary Figure 3a). Closer inspection revealed very similar results to those obtained during the compound presentation: a change across time showing that reward firing decreased across trials for Omission ($F(1,401)=49.5$, $p<0.05$), with the difference between baseline and reward firing during the early trials ($t(25)=4.0$, $p<0.05$) disappearing by the late trials ($t(25)=1.4$, $p>0.05$). A similar change in reward firing across trials was obtained for Overexpectation ($F(1,401)=9.4$, $p<0.05$) but not for Control ($F(1,401)=2.7$, $p<0.05$), while preserving the difference in firing between the baseline and reward period for the latter two conditions during the early and late trials (t's$(25)\geq 7.5$, p's<0.05).

The above changes in neural firing following the presentation of the Overexpectation and Omission compounds are suggestive of the presence of an attentional or perhaps even an error-correcting signal. Such an interpretation would require similar neural firing during the reward period between Overexpectation and Omission, with each differing in the same direction from Control. This was not the case. Further analyses revealed that firing to reward at the start of the phase differed between the conditions ($F(2,50)=58.61$, $p<0.05$, Supplementary Figure 3a) such that firing was greater at time of reward following the Overexpectation compound (vs. Control, $q=3.53$, $p<0.05$) and lower following the Omission compound (vs. Control, $q=9.04$, $p<0.05$). Interestingly, while the latter result is consistent with the difference in reward delivery between Omission (zero pellets delivered) and Control (two pellets delivered), the former difference is striking given that the reward delivered following the presentation of the Overexpectation and Control compound was identical (two pellets in both conditions). The difference between Overexpectation and Control suggests that firing during reward delivery may reflect not only presence of reward but also the expectation of reward for two reasons. Firstly, delivery of reward occurs during the last second of compound presentation. Secondly, the delivery of the first pellet represents the best temporal predictor of the arrival of a second pellet. Critically, the delivery of the second pellet would serve to predict the arrival of

the additional overexpected pellets in the Overexpectation but not in the Control condition. This is supported by the finding that the neural summation seen during the compound predicts the additional expectation of two pellets at time of reward: The difference in firing between the Overexpectation and Control compounds during the early trials is correlated with the same difference during the reward period ($r^2=0.46$, $p<0.05$, Supplementary Figure 3b).

Finally, the difference in firing at time of the reward period between Overexpectation and Control was adjusted: The difference in reward firing following these two compounds disappeared on subsequent days of Compound conditioning (each $q<1$).

SUPPLEMENTARY NOTE 4. Neural firing: Unique populations during Compound Probe.

Two unique neural populations were defined on the basis of showing an increase in firing at time of reward, and a decline in firing between the early and the late trials during the Compound Probe phase for Overexpectation only and for Omission only. These populations showed very similar firing profiles to those obtained for the common population. The Overexpectation-unique population of 11 cells tracked the changes in reward expectancy in Overexpectation. An index of the difference in firing between Overexpectation and Control compounds was computed (Supplementary Figure 4). The distribution of these indices was positively shifted during the early trials ($\mu=0.53$, $t(10)=3.8$, $p<0.05$), but centered on zero during the late trials ($\mu=-0.02$, $t(10)=-0.2$ $p>0.05$), with a significant difference between them ($t(10)=3.9$, $p<0.05$). This shows that firing to the Overexpectation compound was greater compared to the Control compound at the start but equivalent by the end of the Compound Probe phase.

Similarly, 16 cells in the Omission-unique population also tracked changes in reward expectancy. An index was computed representing the difference in firing between Omission and Control (Supplementary Figure 4). The distribution of the indices was centered on zero during the early trials ($\mu=-0.18$, $t(15)=0.9$, $p>0.05$) but negatively shifted during the late trials ($\mu=-0.37$, $t(15)=-2.7$, $p<0.05$), with a

significant difference between them ($t(15)=3.3$, $p<0.05$). Thus, there were no differences in neural firing between the Omission and Control compounds during the early trials, and a clear difference emerged such that neural firing was lower to the Omission compared to the Control compound by the late trials. These analyses show that the population of cells that show a decline in firing uniquely to Overexpectation or uniquely to Omission tracked the change in reward expectancy in the Overexpectation and Omission conditions, respectively.

SUPPLEMENTARY NOTE 5. CN neural firing does not signal changes in attention.

Poor but not good predictors of outcomes command attention[22]. In the present task, both the Overexpectation and Omission compounds were poor predictors of their corresponding outcomes during the Compound Probe phase, whereas the Control compound consistently signaled its predicted outcome. Therefore, it was expected that the Overexpectation and Omission compounds (but not the Control) would each command attention. In order to determine whether CN neurons signaled attention, we screened all 87 cells (irrespective of reward responses) for increase or decreases in neural firing during presentation of the Overexpectation and Omission compounds compared to the Control compound. Greater firing to the Overexpectation compared to the Control compound was obtained in seven neurons (that were not part of the reward-responsive population described in the main text and shown not signal attention) whereas three neurons were shown to exhibit the opposite pattern of results (i.e. lower firing rate, t-test, $p<0.05$). In the case of Omission, seven and one neurons were shown to exhibit greater and lower firing rate compared to the Control compound, respectively (t-test, $p<0.05$). All these cells were below chance (t's$(999)=163.3$, p's<0.05).
